# Frequent epigenetic inactivation of *RASSF2 *in thyroid cancer and functional consequences

**DOI:** 10.1186/1476-4598-9-264

**Published:** 2010-09-29

**Authors:** Undraga Schagdarsurengin, Antje M Richter, Juliane Hornung, Cornelia Lange, Katrin Steinmann, Reinhard H Dammann

**Affiliations:** 1 Institute for Genetics, Justus-Liebig-University Giessen, Germany; 2 Institute of Biochemistry, Medical Faculty, Justus-Liebig-University Giessen, Germany

## Abstract

**Background:**

The Ras association domain family (RASSF) encodes for distinct tumor suppressors and several members are frequently silenced in human cancer. In our study, we analyzed the role of RASSF2, RASSF3, RASSF4, RASSF5A, RASSF5C and RASSF6 and the effectors MST1, MST2 and WW45 in thyroid carcinogenesis.

**Results:**

Frequent methylation of the *RASSF2 *and *RASSF5A *CpG island promoters in thyroid tumors was observed. *RASSF2 *was methylated in 88% of thyroid cancer cell lines and in 63% of primary thyroid carcinomas. *RASSF2 *methylation was significantly increased in primary thyroid carcinoma compared to normal thyroid, goiter and follicular adenoma (0%, 17% and 0%, respectively; p < 0.05). Patients which were older than 60 years were significantly hypermethylated for *RASSF2 *in their primary thyroid tumors compared to those younger than 40 years (90% vs. 38%; p < 0.05). *RASSF2 *promoter hypermethylation correlated with its reduced expression and treatment with a DNA methylation inhibitor reactivated *RASSF2 *transcription. Over-expression of RASSF2 reduced colony formation of thyroid cancer cells. Functionally our data show that RASSF2 interacts with the proapoptotic kinases MST1 and MST2 and induces apoptosis in thyroid cancer cell lines. Deletion of the MST interaction domain of RASSF2 reduced apoptosis significantly (p < 0.05).

**Conclusion:**

These results suggest that *RASSF2 *encodes a novel epigenetically inactivated candidate tumor suppressor gene in thyroid carcinogenesis.

## Background

Approximately 10% of the whole population develop a clinically significant thyroid nodule during their lifetime [1]. Nearly 80% of all thyroid malignancies arise from the follicular epithelial cells, the thyrocytes. There are three main histological types of epithelial thyroid cancer: the papillary thyroid carcinoma (PTC), the follicular thyroid carcinoma (FTC) and the undifferentiated (or anaplastic) thyroid carcinoma (UTC). In contrast, medullary thyroid carcinoma (MTC) develop from parafollicular C cells. C cells produce calcitonin, a hormone regulating the calcium metabolism [2].

Development and progression of thyroid tumors are a consequence of complex epigenetic and genetic changes. In our previous work, we have identified a novel Ras effector, which is located at the tumor suppressor gene locus 3p21.3 [3]. The gene termed *Ras Association Domain Family 1A *(*RASSF1A*) is epigenetically inactivated in human cancers and inhibits the growth of tumors *in vivo *[4,5]. We and others have shown that *RASSF1A *is frequently silenced in thyroid cancer [6-9]. *RASSF1A *hypermethylation was more pronounced in the aggressive undifferentiated thyroid carcinoma [7,10]. Epigenetic inactivation of *RASSF5 *in thyroid cancer has also been investigated [11,12].

RASSF1A was the first member of the Ras association domain family (RASSF). The family consists of six classical RASSFs (RASSF1 to RASSF6) and the N-terminal RASSFs (RASSF7 to RASSF10) with a N-terminal Ras association (RA) domain [13-17]. The classical RASSFs encode a C-terminal RA and SARAH domain [13,16]. Several of these genes (e.g. *RASSF1A, RASSF2, RASSF5 [NORE1], RASSF6*) encode tumor suppressors, which are involved in cell cycle regulation, microtubule stability and in apoptosis [13,16,18-20]. The SARAH domain is a protein-protein interaction domain, named after the tumor suppressors Salvador (in *D. melanogaster*; orthologue of human WW45), RASSF1 and Hippo (in *D. melanogaster*; orthologue of the human proapoptotic kinase MST1). SARAH domains are found in WW45, MST1 and all classical RASSFs. Heteromeric as well as homomeric interactions can be conducted via their SARAH domains [21], e.g. MST and WW45 or RASSF1A and RASSF5 interaction [22-24]. SARAH domains play a central role in the newly discovered Hippo signaling pathway in *D. melanogaster*, which regulates cell proliferation and apoptosis [21,25].

The aim of our study was to reveal the epigenetic status and function of *RASSF2, RASSF3, RASSF4, RASSF5 *and *RASSF6 *in thyroid carcinogenesis. Here, we report that *RASSF2 *is frequently hypermethylated in thyroid tumors and suppresses growth of thyroid cancer.

## Results

### Epigenetic inactivation of *RASSF2 *and *RASSF5 *in thyroid cancer

**Figure 1:**
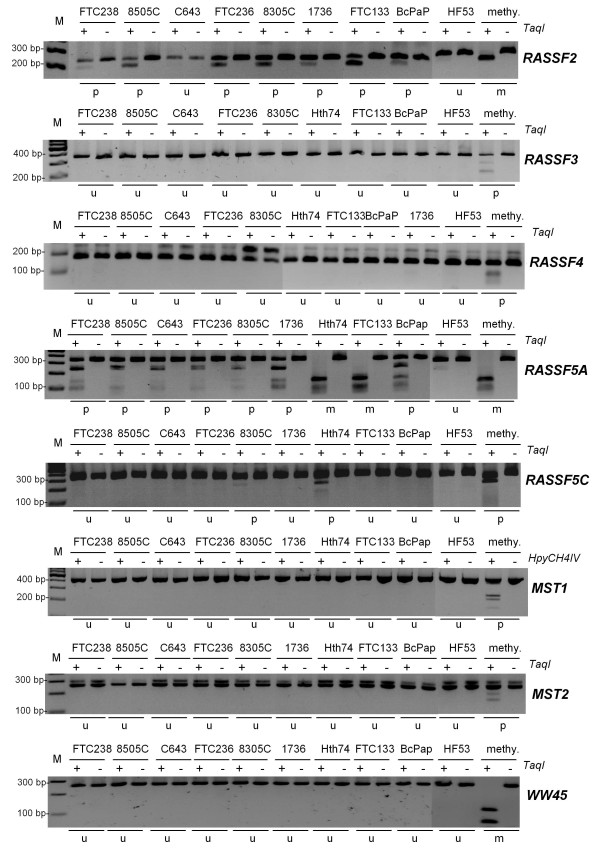
**Methylation of *RASSFs, MSTs *and *WW45 *in thyroid cancer cell lines**. The methylation status of the indicated CpG islands was analyzed by COBRA. PCR products (for length see Additional file 5, Table S1) from bisulfite-treated DNA were digested (+) and mock digested (-) with *TaqI *or *HpyCH4IV *and resolved on 2% gel. *In vitro *methylated DNA (methy.) was utilized as positive control. The sizes of a 100 bp ladder (M) are indicated. Methylated (m), partially methylated (p) and unmethylated (u) samples were determined.

In our study we aimed to reveal the role of the classical RASSF members which harbor a C-terminal RA domain and a SARAH domain and their effectors MST1, MST2 and WW45 in thyroid carcinogenesis. Since *RASSF1A *function and silencing was already studied in detail, we focused our work on RASSF2, RASSF3, RASSF4, RASSF5A, RASSF5C and RASSF6 which all encode a C-terminal SARAH domain [7,10,13]. First we analyzed the promoter methylation of these genes in thyroid cancer cell lines (Fig. 1). *RASSF6 *contains the only promoter, which is not located within a CpG island [13] and was not included in the methylation analysis. *RASSF2 *promoter hypermethyation was observed in seven out of eight (88%) thyroid cancer cell lines (Fig. 1 and Tab. 1). *RASSF5A *hypermethylation was found in all analyzed cancer cell lines, however *RASSF5C *was only partially methylated in 8305C and Hth74 (Fig. 1). *RASSF5A *and *RASSF5C *are transcribed from different CpG island promoters [13]. CpG island hypermethylation of *RASSF2 *and *RASSF5A *was confirmed by bisulfite sequencing (additional file 1). CpG island promoter hypermethylation of *RASSF3*, *RASSF4, MST1, MST2 *and *WW45 *were not found in thyroid cancer cell lines (Fig. 1). *RASSF1A *methylation was analyzed previously and is summarized in Table 1[7,10]. *RASSF1A *was hypermethylated in all analyzed thyroid cancer cell lines.

**Table 1:** Summary of *RASSF2, RASSF5A *and *RASSF1A *methylation analyses

	*RASSF2*	*RASSF5A*	***RASSF1A***^**c**^
thyroid cancer cell lines	7/8 (88%)	9/9 (100%)	9/9 (100%)

primary thyroid cancer	19/30 (63%)^a, b^	26/31 (84%)	26/31 (84%)
MTC	0/3 (0%)	1/3 (33%)	5/6 (83%)
PTC	6/11 (54%)	9/12 (75%)	8/13 (62%)
FTC	8/10 (80%)	10/10 (100%)	7/10 (70%)
UTC	5/6 (83%)	6/6 (100%)	7/9 (77%)

follicular adenoma	0/10 (0%)^a^	10/10 (100%)	7/10 (70%)

goiter	2/12 (17%)^b^	12/12 (100%)	9/12 (75%)

control tissue	0/12 (0%)^a^	12/12 (100%)	-

significant differences: ^a^primary thyroid carcinoma vs. follicular adenoma or primary thyroid carcinoma vs. normal thyroid (p≤0.001, Fisher's exact test); ^b ^primary thyroid carcinoma vs. goiter (p = 0.015, Fisher's exact test); ^c^[7,10].

**Figure 2:**
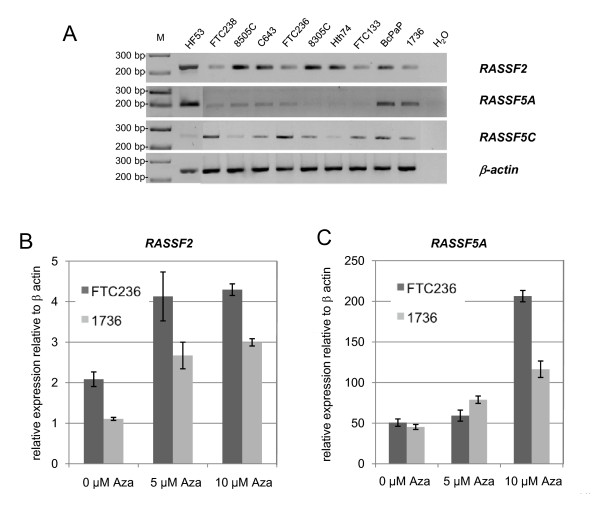
***RASSF2 *and *RASSF5 *expression in thyroid cancer cell lines**. cDNA isolated from thyroid cancer cell lines and human fibroblasts (HF53) were amplified by RT-PCR (for primer and conditions see Additional file 6, Table S2) together with a water control (H_2_O). *PCR *products were resolved on 2% gel together with a 100 bp ladder (M). Expression of β-actin (230 bp) was determined as a control for RNA integrity. **B**. Reexpression of *RASSF2 *was analyzed by qRTPCR in thyroid cancer cell lines FTC133 and 1736 treated for 4 days with indicated concentrations of 5-aza-2'-deoxycytidine (Aza). RNA levels were normalized to β-actin and calculated relative to human fibroblasts (= 100) **C**. Reexpression of *RASSF5A *after Aza treatment.

Next we analyzed the expression of RASSF2, RASSF5A and RASSF5C in all thyroid cancer cell lines and human fibroblasts (HF53) (Fig. 2). In human fibroblasts, which were unmethylated for all three promoters, high expression of *RASSF2 *and *RASSF5A *was detected, however *RASSF5C *expression was low (Fig. 2A). Expression of *RASSF2 *was reduced in several thyroid cancer cell lines (e.g. FTC 236 and 1736). *RASSF5A *expression was also diminished in different thyroid cancer cell lines (Fig. 2A) compared to HF53. Additionally, two thyroid cancer cell lines (FTC 236 and 1736) were treated with 5-Aza-2'-deoxycytidine (Aza) and RASSF2 and RASSF5A expression was analyzed by qRT-PCR (Fig. 2B and 2C). Inhibition of DNA methyltransferases by Aza induced an upregulation of *RASSF2 *and *RASSF5A *expression. In summary, we observed frequent epigenetic silencing of *RASSF2 *and *RASSF5A *in thyroid cancer cell lines.

### Frequent tumor-specific methylation of RASSF2 in primary thyroid tumors

**Figure 3:**
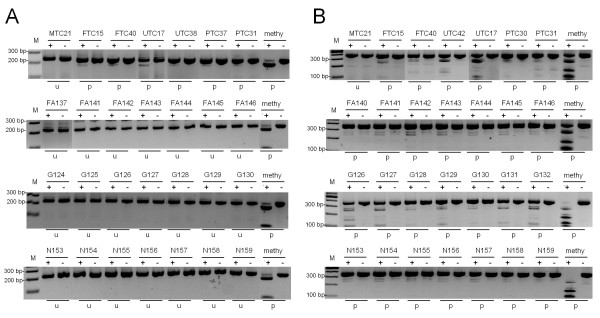
**Methylation of *RASSF2 *and *RASSF5A *in primary tissue**. **A**. COBRA of RASSF2 in primary thyroid specimen, including follicular thyroid cancer (FTC), papillary thyroid cancer (PTC), medullary thyroid cancer (MTC), undifferentiated thyroid cancer (UTC), goiter (G) normal thyroid samples (N) and follicular adenoma (FA). PCR products from bisulfite-treated DNA were digested (+) and mock digested (-) with *TaqI *and resolved on 2% gel. **B**. Methylation status of *RASSF5A*. *In vitro *methylated DNA (methy.) was utilized as positive control. The sizes of a 100 bp ladder (M) are indicated. Methylated (m), partially methylated (p) and unmethylated (u) samples were determined.

Subsequently, we analyzed the methylation of *RASSF2 *and *RASSF5A *promoter in 31 primary thyroid carcinomas (3 MTC, 10 FTC, 12 PTC and 6 UTC), 10 follicular adenomas, 12 goiters and 12 normal thyroid controls (Fig. 3 and Tab. 1). In 19 out of 30 (63%) thyroid carcinomas, the promoter of *RASSF2 *was partially methylated (Tab. 1). In contrast, none of 12 normal thyroid tissues and 10 follicular adenomas exhibited a methylation of *RASSF2 *(Fig. 3A). Only two out of 12 (17%) goiters were methylated (Tab. 1). Statistical analysis revealed a significant increase in *RASSF2 *methylation frequency in thyroid cancer compared to normal thyroid controls, follicular adenomas or goiters (p < 0.001, p = 0.001 or p = 0.015; Fisher's exact test, respectively). Furthermore, we examined the methylation frequency of *RASSF2 *in different types of thyroid carcinoma (Tab. 1). Medullary thyroid carcinoma showed the lowest frequency (0/3 = 0%) in comparison to PTC (6/11 = 54%), to FTC (8/10 = 80%) and to UTC (5/6 = 83%). Methylation of *RASSF2 *was significantly higher in thyroid tumors obtained from older patients (90%; >60 year) compared to those from younger patients (38%; <40 years; p = 0.043) or patients from 41 to 59 years (58%). Other correlations with histopathological parameters were not revealed. We also analyzed the *RASSF5A *methylation in primary thyroid cancers and other thyroid tissues (Fig. 3B). *RASSF5A *methylation was frequently found in cancer cell lines (9/9 = 100%) and primary thyroid cancer but was also detected in all analyzed control tissues, goiters and follicular adenomas (Tab. 1). Since RASSF5A methylation was not tumor specific, we further focused on RASSF2 where methylation was preferentially found in cancers.

### Functional analysis of RASSF2

**Figure 4:**
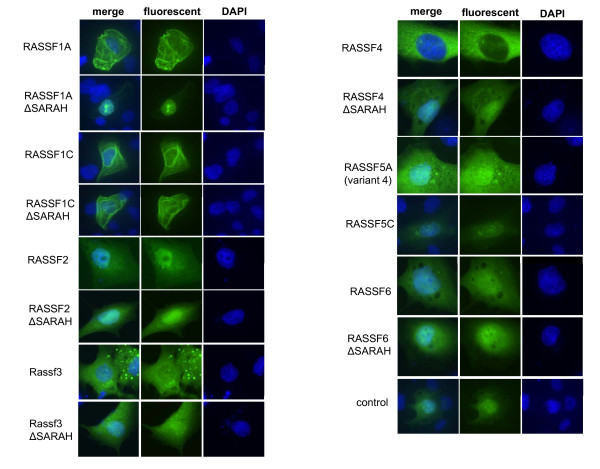
**Localization of fluorescent RASSFs and RASSFΔSARAHs (deletion of SARAH domain)**. FTC133 cells were transfected with the indicated fluorescent RASSFs and control vector (pEGFP). After one day cells were fixed and stained with DAPI.

**Figure 5:**
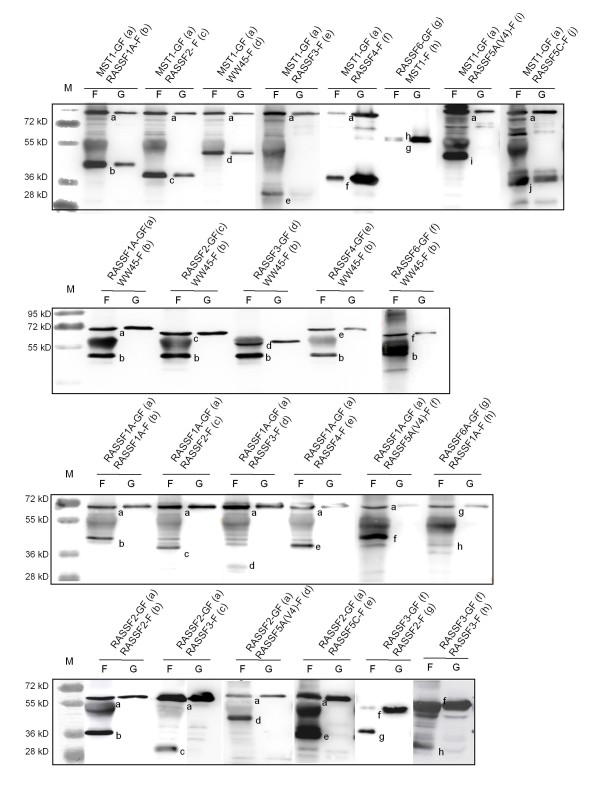
**Binding studies of RASSFs, MST1 and WW45 in co-precipitation**. Plasmids, that express the indicated GST-Flag-tagged-constructs (-GF) and Flag-tagged-constructs (-F) were transfected into HEK293T cells. After two days, total protein was extracted and Flag-tagged proteins were precipitated with anti-Flag-agarose (F) or glutathione sepharose (G). Samples were separated on a 10% PAGE gel and blotted. The precipitated and co-precipitated proteins were determined with anti-Flag-antibodies. The sizes of a protein marker (M) are indicated.

Next, we analyzed the distinct tumor suppressive features associated with RASSF2. First, the localization of RASSF2 and the other classical RASSFs was investigated in FTC133 cells (Fig. 4). RASSF1A and RASSF1C are both found in the cytoplasm together with microtubule. The other RASSFs (RASSF2, Rassf3, RASSF4, RASSF5 and RASSF6) were not associated with microtubule but were observed in the cytoplasm and nucleus (Fig. 4 and data not shown). For Rassf3, RASSF4, RASSF5 and RASSF6 cluster formation was detected (Fig. 4 and data not shown). Deletion of the SARAH domain did not alter the localization of the fluorescent RASSF2, Rassf3 and RASSF6. However RASSF1A and RASSF4 appear to localize to nucleus from cytoplasm. It is interesting to note that the utilized RASSF5A isoform (variant 4) contains a partially truncated SARAH domain. The SARAH domain is a protein-protein interaction domain, which is found in all classical RASSFs and their proapoptotic effector MST1 and MST2 and the adapter protein WW45. Therefore, we analyzed the interaction of these proteins in yeast two hybrid and pull down assays (Table 2 and Fig. 5). We found an interaction of all RASSFs with MST1 and MST2 in the yeast two hybrid assay (additional file 2). However, truncation of the SARAH of RASSF5A (variant 4) and RASSF2 reduced the interaction in the yeast two hybrid or abolished interaction in the pulldown assay (Fig. 5, additional file 2 and additional file 3). Interaction of WW45 with RASSF1A, RASSF3, RASSF4 and MST1 and MST2 in the yeast two hybrid was found. Precipitation of MST1 with WW45, RASSF1A, RASSF2, RASSF3, RASSF5C and RASSF6 was also confirmed by pull down (Tab. 2 and Fig. 5). Interaction of RASSF2 with RASSF1A, RASSF3 and RASSF5C was only found in yeast two hybrid assay (Tab. 2).

**Table 2:** Summary of interaction analyses

interaction partners		interaction in Y2H	interaction in pulldown
MST1/MST2	RASSF1A	+/+	+/na
	RASSF2	+/+	+/na
	RASSF3	+/+	+/na
	RASSF4	+/+	+/na
	RASSF5A(V4)	w/w	-/na
	RASSF5C	+/+	+/na
	RASSF6	+/+	+/na
	WW45	+/+	+/na

WW45	RASSF1A	w	-
	RASSF2	-	-
	RASSF3	w	-
	RASSF4	w	-
	RASSF5A(V4)	w	na
	RASSF6	-	-

RASSF1A	RASSF1A	+	-
	RASSF2	-	-
	RASSF3	-	-
	RASSF4	-	-
	RASSF5A(V4)	-	-
	RASSF6	-	-

RASSF2	RASSF1A	w	-
	RASSF2	-	-
	RASSF3	+	-
	RASSF4	-	-
	RASSF5A(V4)	-	-
	RASSF6	-	-

RASSF3	RASSF1A	-	-
	RASSF2	-	-
	RASSF3	w	w
	RASSF4	+	-
	RASSF5A(V4)	-	na
	RASSF6	-	-

RASSF4	RASSF1A	-	-
	RASSF2	-	-
	RASSF3	+	-
	RASSF4	-	-
	RASSF5A(V4)	-	-
	RASSF6	-	-

RASSF5C	RASSF1A	+	w
	RASSF2	+	-
	RASSF3	-	na
	RASSF4	w	-
	RASSF5A(V4)	-	na
	RASSF6	-	

RASSF6	RASSF1A	-	-
	RASSF2	-	-
	RASSF3	-	-
	RASSF4	-	-
	RASSF5A(V4)	-	-
	RASSF6	-	na

+, positive interaction; -, no interaction; w, weak interaction; na, not analyzed.

### RASSF2-induced growth suppression and apoptosis in thyroid cancer

**Figure 6:**
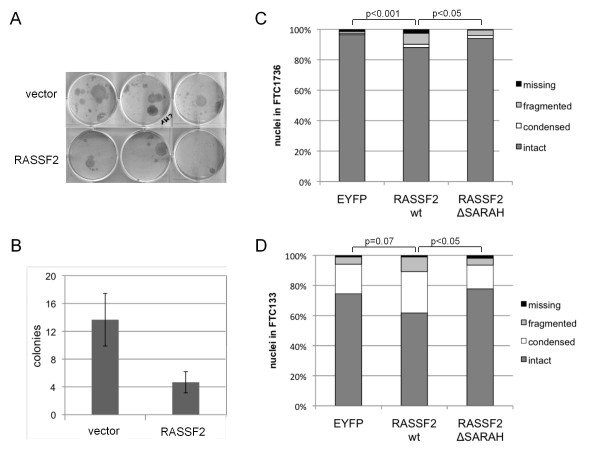
**Tumor suppressor function of RASSF2**. **A**. The *RASSF2 *was cloned in pCMV-Tag1 and transfected in thyroid cancer cell line 1736 in parallel with empty vector in triplicates. Cells were grown in selective medium for four weeks and stained with Giemsa. **B**. Numbers of colonies were determined and means with standard deviation were plotted. **C**. 1736 cells were transfected with the indicated fluorescent RASSF2 constructs and control vector (pEYFP). After two days cells were fixed and stained with DAPI. Fluorescent cells (n > 200) were counted and the percentage of intact, condensed, fragmented and missing nuclei were determined. **D**. Nuclei alterations in FTC133 cells (n > 100).

The interaction of RASSF2 with kinases MST1 and MST2 indicate that RASSF2 is involved in the proapoptotic RASSF-pathway. Therefore, we analyzed RASSF2-induced growth suppression and apoptosis in thyroid cancer cell lines (Fig. 6). The thyroid cancer cell line 1736 was transfected with a construct expressing *RASSF2 *under control of the CMV promoter or an empty vector (Fig. 4). After selection for 4 weeks a 65% decrease of colony formation in cancer cells transfected with *RASSF2 *(mean = 4.6) compared to cells transfected with the empty vector (mean = 13.6; p = 0.019, one-way ANOVA test) was observed (Fig. 6A and 6B). Additionally, we analyzed the RASSF2 induced apoptosis in 1736 and FTC133 cells (Fig. 6C and 6D; additional file 4). Transfection of fluorescent RASSF2 caused a significantly increased apoptosis indicated by nuclei condensation/fragmentation compared to the fluorescent vector in 1736 and FTC133 (p < 0.001 and p = 0.07, respectively). Apoptotic cells were exemplarily verified by TUNEL assay (Fig. S4). In FTC1736 the ratio of intact vs. apoptotic cells was for the vector: 96% vs. 4%; for RASSF2wt: 88% vs. 12% and for RASSF2ΔSARAH: 94% vs. 6%, respectively. In FTC133 a similar trend was observed (Fig. 6D). For FTC133 the ratio of intact vs. apoptotic cells was the following for the vector: 75% vs. 25%; for RASSF2wt: 62% vs. 38% and for RASSF2ΔSARAH: 78% vs. 22%, respectively. Thus, RASSF2ΔSARAH exhibited a significantly reduced rate of apoptotic cells in 1736 and FTC133 cells compared to wt RASSF2 (p < 0.05). In summary, our data suggest that RASSF2 tumor suppression function involves apoptotic regulation through binding of the proapoptotic kinases MST.

## Discussion

Epigenetic inactivation of tumor suppressor genes is a fundamental event in the pathogenesis of malignant tumors. Here we report that *RASSF2*, a member of the Ras Association Domain family, is frequently silenced in primary thyroid tumor and thyroid cancer cell lines. To the best of our knowledge this is the first report that shows that the epigenetic inactivation of *RASSF2 *is a common event in thyroid carcinogenesis. We also show that RASSF2 suppressed growth of thyroid cancer cells. The *RASSF2 *transcript can be detected in most normal tissues [20]. Down-regulation of *RASSF2A *by promoter hypermethylation has been shown in different tumor cell lines and primary tumors, including lung cancer, colon cancer, gastric cancer, nasopharyngeal carcinoma, head and neck cancer [20,26-33]. It has been reported that primary colorectal cancers, which showed *KRAS *or *BRAF *mutations, also frequently showed *RASSF2 *methylation, and inactivation of *RASSF2 *enhanced KRAS-induced oncogenic transformation [28,30]. RASSF2 binds directly to K-Ras in a GTP-dependent manner via the Ras effector domain [20]. In addition, patients with methylated *RASSF2A *promoter presented a higher frequency of lymph node metastasis [27]. Methylation of *RASSF1A *and *RASSF2 *was associated with poor outcome after radiotherapy in oral squamous cell carcinoma [34]. Here we report that the degree of *RASSF2 *methylation was associated with the age of thyroid cancer patients. It has been suggested that aberrant methylation of *RASSF2 *in plasma of colorectal cancer patients may serve as a cancer biomarker [35]. Thus, it will be interesting to determine hypermethylation of *RASSF2 *in blood samples of thyroid cancer patients.

Regarding RASSF2's interaction with other RASSF members, it was shown to associate with RASSF3 and RASSF5 [29]. Our yeast two hybrid assay confirmed these bindings and additionally binding of RASSF1A and RASSF2 was observed. However these interactions were not observed in pulldown (Tab. 2). In general only the strong yeast two hybrid interactions could be confirmed by pulldowns. Since yeast two hybrid assays are prone to false positive results, we rather agree with the pull down interactions. RASSF2 also binds both proapoptotic kinases MST1 and MST2 in yeast two hybrid assays and this was confirmed for RASSF2 and MST1 by pulldown. It has been suggested that binding of the classical RASSFs, MST1, MST2 and WW45 with each other is conducted by their SARAH domains [21]. Deletion of the SARAH domain of RASSF2 abolished binding with MST1 and MST2 [36,37]. It is interesting to note that all RASSFs and WW45 bind MST1, however only certain RASSFs (e.g. RASSF1A and RASSF5C) bind each other in pulldowns. This result suggests that SARAH domains show different binding properties.

RASSF2 exhibits several tumor suppressor properties, like inhibition of cell growth and induction of apoptosis [20,27]. Here we show that RASSF2 also acts as a novel tumor suppressor in thyroid carcinogenesis. It has been shown that RASSF2 binds and regulates the proapoptotic kinases MST1 and MST2 [36,37]. RASSF2 also engages the JNK pathway and induces apoptosis in an MST1-independent manner [37]. Other findings suggest that MST2 and RASSF2 form a complex, in which RASSF2 is maintained in a phosphorylated state by MST2 and protects MST2 from degradation and turnover [36]. However, the exact phosphorylation site of RASSF2 by MST2 was not revealed.

Additionally, we have found that *RASSF5A *is frequently hypermethylated in thyroid tissues, however methylation was not tumor specific since it was also observed in controls. Epigenetic silencing of *RASSF5 *in thyroid cancer has been analyzed previously, but was also not tumor specific [11,12]. Interestingly, *RASSF5A *and *RASSF2 *methylation was reduced in MTC compared to other cancers. It has been reported that RASSF5A expression is suppressed in FTC carrying PAX8-PPARγ fusions [11]. Methylation of the other investigated CpG island promoters of RAS effectors (*RASSF3, RASSF4*, *MST1*, *MST2 *and *WW45*) was uncommon.

## Conclusions

In summary, we report frequent tumor-specific hypermethylation of *RASSF2 *in thyroid cancer. Functional analysis showed that RASSF2 interacts with both proapoptotic kinases MST1 and MST2. Overexpression of RASSF2 induces apoptosis in thyroid cancer cell lines. These data suggest that RASSF2 acts as a proapoptotic tumor suppressor in thyroid carcinogenesis.

## Methods

### Tissues and cell lines

65 thyroid tissues, including 31 primary thyroid carcinomas (6 UTC, 3 MTC, 10 FTC, and 12 PTC), 10 follicular adenomas, 12 goiters and 12 normal thyroid samples were analyzed [7,10]. All patients signed informed consent at initial clinical investigation. The study was approved by local ethic committees. The mean age of the study population at the time of surgical resection was 51 ± 15 years and 40% of the patients were male. Nine human thyroid cancer cell lines: BcPaP (PTC), FTC238, FTC236, FTC133, C643 (UTC), 8505C (UTC), 1736 (UTC), 8305C (UTC) and HTh74 (UTC) were cultured in the recommended medium and included in this study [7,10]. Genomic DNA was extracted from frozen tissues and cultured cells by a standard phenol/chloroform procedure.

### Methylation analysis

Methylation of promoter regions (*RASSF2*, *RASSF3*, *RASSF4*, *RASSF5A*, *RASSF5C*, *WW45*, *MST1 *and *MST2*) was determined by COBRA and bisulfite sequencing [38,39]. Briefly, 100 ng of bisulfite-treated DNA were amplified with 10 pmol of primers in a reaction buffer containing 0.2 mM dNTP mix, 1.5 mM MgCl_2_, 10 pmol of each primer and 1.5 U Taq polymerase (InViTek GmBH, Berlin, Germany). For PCR primers and condition see additional file 5. 20 to 50 ng of PCR products were digested with 10 U of *TaqI *or HpyCH4IV (NEB, Frankfurt, Germany) and analyzed on a 2% Tris-borate EDTA agarose gel. For bisulfite sequencing PCR products were cloned in the pGEM-T vector (Promega, Heidelberg, Germany) and sequenced (Genterprise, Mainz, Germany).

### Expression analysis

Two thyroid cancer cell lines (1736 and FTC236) were treated for 4 days with 5 μM or 10 μM of 5-aza-dC (Sigma, Taufkirchen, Germany). RNA was isolated using TRIzol-Reagent (Invitrogen, Karlsruhe, Germany). To eliminate DNA contamination 1 μg RNA was incubated with 1U DNase I (Fermentas GmbH, St. Leon-Rot, Germany), 1 μl 10X DNase I buffer and DEPC-treated water in 10 μl. After 15 minutes incubation at room temperature DNase was inactivated by adding 1 μl of 25 mM EDTA and incubation at 65°C for 15 min and reversely transcribed using poly-dT primers and random hexamers in 20 μl of RT-mix with MMLV reverse transcriptase (Promega, Heidelberg, Germany) for 1 h at 42°C. Subsequently, 2 μl of cDNA was amplified. For PCR primers and condition see additional file 6.

### Statistical evaluation

Statistical analysis was carried out using SPSS17 (SPSS, München, Germany). Categorical variables were plotted into contingency tables and evaluated using Fisher's exact test or one way ANOVA test. All reported p-values are two-sided and considered significant for p < 0.05.

### Constructs

The cDNAs of *RASSF2*, *RASSF3*, *RASSF4*, *RASSF5A *(variant 4), *RASSF5C *(variant 3), *RASSF6*, *MST1*, *MST2 *and *WW45 *were cloned after amplification of fragments from EST-clones DKFZp781O1747Q, IMAGp998A0813502Q, IRAKp961I1269Q, IRALp962N0113Q (BC004270), IRAKp961L2026Q; DKFZp686K23225Q, IRAKp961C0282Q, IRAKp961I0613Q and IRAKp961L0427Q (RZPD, Berlin, Germany), respectively. RASSF1A, RASSF1C and Rassf3 were described previously [3,40]. Mutant forms of constructs were generated with the QuickChange^® ^XL Site-Directed Mutagenesis Kit (Stratagene, La Jolla, USA) and appropriate primers. All constructs were confirmed by sequencing.

### Localization studies

cDNAs were cloned into the fluorescence vector pEYFP (Clontech, Mountain View, USA). After transient transfection into FTC133 cells with HEKfectin (BioRad, München, Germany), the localization of fluorescent RASSF variants and the vector control were investigated with a fluorescence microscope (Axio Observer Z1 Zeiss, Jena, Germany). Nuclei of the cells were visualized by staining with DAPI (0,1 μg/ml in PBS).

### Interaction studies using the yeast two-hybrid system

The Matchmaker Two-hybrid system (Clontech, Mountain View, USA) was utilized. The yeast strain PJ69-4A was co-transformed with 0.5 μg of each plasmid using the PEG/LiAc method. The interaction analysis was carried out on SD minimal medium plates without adenine and histidine and the transformation efficiency was determined on SD plates with adenine and histidine. The strength of interaction was investigated by quantification of the expression of the β-galactosidase reporter gene with o-nitrophenyl-β-D-galactopyranoside (ONPG) as substrate at OD 420 nm.

### Interaction studies by co-precipitation

cDNAs were cloned into the vector pCMV-Tag1 (Stratagene, La Jolla, USA) and/or in the modified vector pEBG (GST and FLAG tag). To investigate the interaction of specific RASSF forms, MST1, MST2 and WW45, co-transfections with HEKfectin (BioRad, München, Germany), were performed in HEK293T cells. Two days after transfection, total protein was extracted in FLAG-lysis buffer. The GST-fused proteins were precipitated with glutathione-sepharose (Amersham Biosciences, Freiburg, Germany). FLAG-tagged proteins were precipitated with anti-Flag-agarose (Sigma, Steinheim, Germany). Samples were separated on a 10% PAGE gel and blotted. The interaction was determined with anti-FLAG-antibodies (Sigma, Steinheim, Germany).

### Proliferation assays

RASSF2 was cloned into the vector pCMV-Tag1 (Stratagene, La Jolla, USA). The thyroid cancer cell line 1736 was transfected using HEKfectin (BioRad, München, Germany). Colonies were selected under 1 mg/ml Geneticin (Gibco, Karlsruhe, Germany) in DMEM for 4 weeks. Expression of RASSF2 was confirmed by RT-PCR (data not shown).

### Apoptotic assays

Thyroid cancer cells FTC133 and 1736 were seeded on glass slides and transfected with different RASSF2 constructs using HEKfectin (BioRad, München, Germany) and analyzed after two days of incubation. Therefore cells were fixed with paraformaldehyde, permeabilized with TritonX, DAPI stained and embedded in MOWIOL for fluorescent analysis. Transfected cells (n > 100 for FTC133 and n > 200 for 1736) were analyzed for intact, condensed, fragmented or missing nucleus.

## Abbreviations

RASSF: Ras association domain family; MST: mammalian sterile 20-like kinase/human Hippo homolog; WW45: human Salvador homolog; SARAH: Salvador-RASSF-Hippo; COBRA: combined bisulfite restriction analysis; 5-AZA-DC: 5-aza-2'-deoxycytidine; UTC: undifferentiated thyroid carcinoma; PTC: papillary thyroid carcinoma; MTC: medullary thyroid carcinoma; FTC: follicular thyroid carcinoma.

## Competing interests

The authors declare that they have no competing interests.

## Authors' contributions

RHD has created the study. AMR, US, KS and RHD participated in the design of the study. AMR, US, CL, JH and KS acquired data. AMR, US, CL, JH and RHD controlled, analyzed and interpreted data. AMR, US, CL, JH and RHD prepared the manuscript. All authors read, corrected and approved the final manuscript.

## Supplementary Material

Additional file 1 **Summary of bisulfite sequencing of *RASSF2 *and *RASSF5A***. Figure with the results of the bisulfite sequencing Click here for file

Additional file 2 **Quantitative interaction analysis in the ONPG assay**. Graph of the quantitative yeast two-hybrid interaction results Click here for file

Additional file 3 **Binding analysis of RASSF2ΔSARAH and MST1 in co-precipitation**. Figure of Western blot Click here for file

Additional file 4 **RASSF2 induced apoptosis**. Figure of TUNEL assay. Click here for file

Additional file 5 **Primer sequences and conditions for COBRA**. Table of oligonucleotides used for methylation analysis Click here for file

Additional file 6 **Primer sequences and conditions for qRT-PCR**. Table of oligonucleotides used for expression analysis Click here for file

## References

[B1] Gimm O Thyroid cancer Cancer Lett 2001 163 143 156 10.1016/s0304-3835(00)00697-2 11165748

[B2] Roman S Mehta P Sosa JA Medullary thyroid cancer: early detection and novel treatments Curr Opin Oncol 2009 21 5 10 10.1097/CCO.0b013e32831ba0b3 19125012

[B3] Dammann R Li C Yoon JH Chin PL Bates S Pfeifer GP Epigenetic inactivation of a RAS association domain family protein from the lung tumour suppressor locus 3p21.3 Nat Genet 2000 25 315 319 10.1038/77083 10888881

[B4] Agathanggelou A Cooper WN Latif F Role of the Ras-association domain family 1 tumor suppressor gene in human cancers Cancer Res 2005 65 3497 3508 10.1158/0008-5472.CAN-04-4088 15867337

[B5] Dammann R Schagdarsurengin U Seidel C Strunnikova M Rastetter M Baier K Pfeifer GP The tumor suppressor RASSF1A in human carcinogenesis: an update Histol Histopathol 2005 20 645 663 10.14670/HH-20.645 15736067

[B6] Hoque MO Rosenbaum E Westra WH Xing M Ladenson P Zeiger MA Sidransky D Umbricht CB Quantitative assessment of promoter methylation profiles in thyroid neoplasms J Clin Endocrinol Metab 2005 90 4011 4018 10.1210/jc.2005-0313 15840741

[B7] Schagdarsurengin U Gimm O Hoang-Vu C Dralle H Pfeifer GP Dammann R Frequent epigenetic silencing of the CpG island promoter of RASSF1A in thyroid carcinoma Cancer Res 2002 62 3698 3701 12097277

[B8] Wong IH Chan J Wong J Tam PK Ubiquitous aberrant RASSF1A promoter methylation in childhood neoplasia Clin Cancer Res 2004 10 994 1002 10.1158/1078-0432.ccr-0378-3 14871978

[B9] Xing M Cohen Y Mambo E Tallini G Udelsman R Ladenson PW Sidransky D Early occurrence of RASSF1A hypermethylation and its mutual exclusion with BRAF mutation in thyroid tumorigenesis Cancer Res 2004 64 1664 1668 10.1158/0008-5472.can-03-3242 14996725

[B10] Schagdarsurengin U Gimm O Dralle H Hoang-Vu C Dammann R CpG island methylation of tumor-related promoters occurs preferentially in undifferentiated carcinoma Thyroid 2006 16 633 642 10.1089/thy.2006.16.633 16889486

[B11] Foukakis T Au AY Wallin G Geli J Forsberg L Clifton-Bligh R Robinson BG Lui WO Zedenius J Larsson C The Ras effector NORE1A is suppressed in follicular thyroid carcinomas with a PAX8-PPARgamma fusion J Clin Endocrinol Metab 2006 91 1143 1149 10.1210/jc.2005-1372 16352687

[B12] Nakamura N Carney JA Jin L Kajita S Pallares J Zhang H Qian X Sebo TJ Erickson LA Lloyd RV RASSF1A and NORE1A methylation and BRAFV600E mutations in thyroid tumors Lab Invest 2005 85 1065 1075 10.1038/labinvest.3700306 15980887

[B13] Richter AM Pfeifer GP Dammann RH The RASSF proteins in cancer; from epigenetic silencing to functional characterization Biochim Biophys Acta 2009 1796 114 128 10.1016/j.bbcan.2009.03.004 19344752

[B14] Sherwood V Manbodh R Sheppard C Chalmers AD RASSF7 is a member of a new family of RAS association domain-containing proteins and is required for completing mitosis Mol Biol Cell 2008 19 1772 1782 10.1091/mbc.E07-07-0652 PMC2291435 18272789

[B15] Sherwood V Recino A Jeffries A Ward A Chalmers AD The N-terminal RASSF family: a new group of Ras-association-domain-containing proteins, with emerging links to cancer formation Biochem J 2010 425 303 311 10.1042/BJ20091318 20025613

[B16] van der Weyden L Adams DJ The Ras-association domain family (RASSF) members and their role in human tumourigenesis Biochim Biophys Acta 2007 1776 58 85 10.1016/j.bbcan.2007.06.003 PMC2586335 17692468

[B17] Schagdarsurengin U Richter AM Wohler C Dammann RH Frequent epigenetic inactivation of RASSF10 in thyroid cancer Epigenetics 2009 4 571 576 10.4161/epi.4.8.10056 19934646

[B18] Allen NP Donninger H Vos MD Eckfeld K Hesson L Gordon L Birrer MJ Latif F Clark GJ RASSF6 is a novel member of the RASSF family of tumor suppressors Oncogene 2007 26 6203 6211 10.1038/sj.onc.1210440 17404571

[B19] Khokhlatchev A Rabizadeh S Xavier R Nedwidek M Chen T Zhang XF Seed B Avruch J Identification of a novel Ras-regulated proapoptotic pathway Curr Biol 2002 12 253 265 10.1016/s0960-9822(02)00683-8 11864565

[B20] Vos MD Ellis CA Elam C Ulku AS Taylor BJ Clark GJ RASSF2 is a novel K-Ras-specific effector and potential tumor suppressor J Biol Chem 2003 278 28045 28051 10.1074/jbc.M300554200 12732644

[B21] Scheel H Hofmann K A novel interaction motif, SARAH, connects three classes of tumor suppressor Curr Biol 2003 13 R899 900 10.1016/j.cub.2003.11.007 14654011

[B22] Ortiz-Vega S Khokhlatchev A Nedwidek M Zhang XF Dammann R Pfeifer GP Avruch J The putative tumor suppressor RASSF1A homodimerizes and heterodimerizes with the Ras-GTP binding protein Nore1 Oncogene 2002 21 1381 1390 10.1038/sj.onc.1205192 11857081

[B23] Chan EH Nousiainen M Chalamalasetty RB Schafer A Nigg EA Sillje HH The Ste20-like kinase Mst2 activates the human large tumor suppressor kinase Lats1 Oncogene 2005 24 2076 2086 10.1038/sj.onc.1208445 15688006

[B24] Hwang E Ryu KS Paakkonen K Guntert P Cheong HK Lim DS Lee JO Jeon YH Cheong C Structural insight into dimeric interaction of the SARAH domains from Mst1 and RASSF family proteins in the apoptosis pathway Proc Natl Acad Sci USA 2007 104 9236 9241 10.1073/pnas.0610716104 PMC1890478 17517604

[B25] Udan RS Kango-Singh M Nolo R Tao C Halder G Hippo promotes proliferation arrest and apoptosis in the Salvador/Warts pathway Nat Cell Biol 2003 5 914 920 10.1038/ncb1050 14502294

[B26] Endoh M Tamura G Honda T Homma N Terashima M Nishizuka S Motoyama T RASSF2, a potential tumour suppressor, is silenced by CpG island hypermethylation in gastric cancer Br J Cancer 2005 93 1395 1399 10.1038/sj.bjc.6602854 PMC2361541 16265349

[B27] Zhang Z Sun D Van do N Tang A Hu L Huang G Inactivation of RASSF2A by promoter methylation correlates with lymph node metastasis in nasopharyngeal carcinoma Int J Cancer 2007 120 32 38 10.1002/ijc.22185 17013896

[B28] Akino K Toyota M Suzuki H Mita H Sasaki Y Ohe-Toyota M Issa JP Hinoda Y Imai K Tokino T The Ras effector RASSF2 is a novel tumor-suppressor gene in human colorectal cancer Gastroenterology 2005 129 156 169 10.1053/j.gastro.2005.03.051 16012945

[B29] Hesson LB Wilson R Morton D Adams C Walker M Maher ER Latif F CpG island promoter hypermethylation of a novel Ras-effector gene RASSF2A is an early event in colon carcinogenesis and correlates inversely with K-ras mutations Oncogene 2005 24 3987 3994 10.1038/sj.onc.1208566 15806169

[B30] Park HW Kang HC Kim IJ Jang SG Kim K Yoon HJ Jeong SY Park JG Correlation between hypermethylation of the RASSF2A promoter and K-ras/BRAF mutations in microsatellite-stable colorectal cancers Int J Cancer 2007 120 7 12 10.1002/ijc.22276 17013898

[B31] Steinmann K Sandner A Schagdarsurengin U Dammann RH Frequent promoter hypermethylation of tumor-related genes in head and neck squamous cell carcinoma Oncol Rep 2009 22 1519 1526 10.3892/or_00000596 19885608

[B32] Kaira K Sunaga N Tomizawa Y Yanagitani N Ishizuka T Saito R Nakajima T Mori M Epigenetic inactivation of the RAS-effector gene RASSF2 in lung cancers Int J Oncol 2007 31 169 173 17549418

[B33] Cooper WN Dickinson RE Dallol A Grigorieva EV Pavlova TV Hesson LB Bieche I Broggini M Maher ER Zabarovsky ER Epigenetic regulation of the ras effector/tumour suppressor RASSF2 in breast and lung cancer Oncogene 2008 27 1805 1811 10.1038/sj.onc.1210805 PMC2948550 17891178

[B34] Huang KH Huang SF Chen IH Liao CT Wang HM Hsieh LL Methylation of RASSF1A, RASSF2A, and HIN-1 is associated with poor outcome after radiotherapy, but not surgery, in oral squamous cell carcinoma Clin Cancer Res 2009 15 4174 4180 10.1158/1078-0432.CCR-08-2929 19509163

[B35] Lee BB Lee EJ Jung EH Chun HK Chang DK Song SY Park J Kim DH Aberrant methylation of APC, MGMT, RASSF2A, and Wif-1 genes in plasma as a biomarker for early detection of colorectal cancer Clin Cancer Res 2009 15 6185 6191 10.1158/1078-0432.CCR-09-0111 19773381

[B36] Cooper WN Hesson LB Matallanas D Dallol A von Kriegsheim A Ward R Kolch W Latif F RASSF2 associates with and stabilizes the proapoptotic kinase MST2 Oncogene 2009 28 2988 2998 10.1038/onc.2009.152 PMC2829092 19525978

[B37] Song H Oh S Oh HJ Lim DS Role of the tumor suppressor RASSF2 in regulation of MST1 kinase activity Biochem Biophys Res Commun 2010 391 969 973 10.1016/j.bbrc.2009.11.175 19962960

[B38] Dammann R Yang G Pfeifer GP Hypermethylation of the cpG island of Ras association domain family 1A (RASSF1A), a putative tumor suppressor gene from the 3p21.3 locus, occurs in a large percentage of human breast cancers Cancer Res 2001 61 3105 3109 11306494

[B39] Xiong Z Laird PW COBRA: a sensitive and quantitative DNA methylation assay Nucleic Acids Res 1997 25 2532 2534 10.1093/nar/25.12.2532 PMC146738 9171110

[B40] Tommasi S Dammann R Jin SG Zhang Xf XF Avruch J Pfeifer GP RASSF3 and NORE1: identification and cloning of two human homologues of the putative tumor suppressor gene RASSF1 Oncogene 2002 21 2713 2720 10.1038/sj.onc.1205365 11965544

