# Supplementary material for: Frequent epigenetic inactivation of RASSF2 in thyroid cancer and functional consequences
Source: Mol Cancer. 2010 Sep 29;9:264. doi: 10.1186/1476-4598-9-264 (PMC2956732; doi:10.1186/1476-4598-9-264)
Supplement: Additional file 1 — Summary of bisulfite sequencing of RASSF2 and RASSF5A. Figure with the results of the bisulfite sequencing [file 1476-4598-9-264-S1.PPT]

## Slide 1
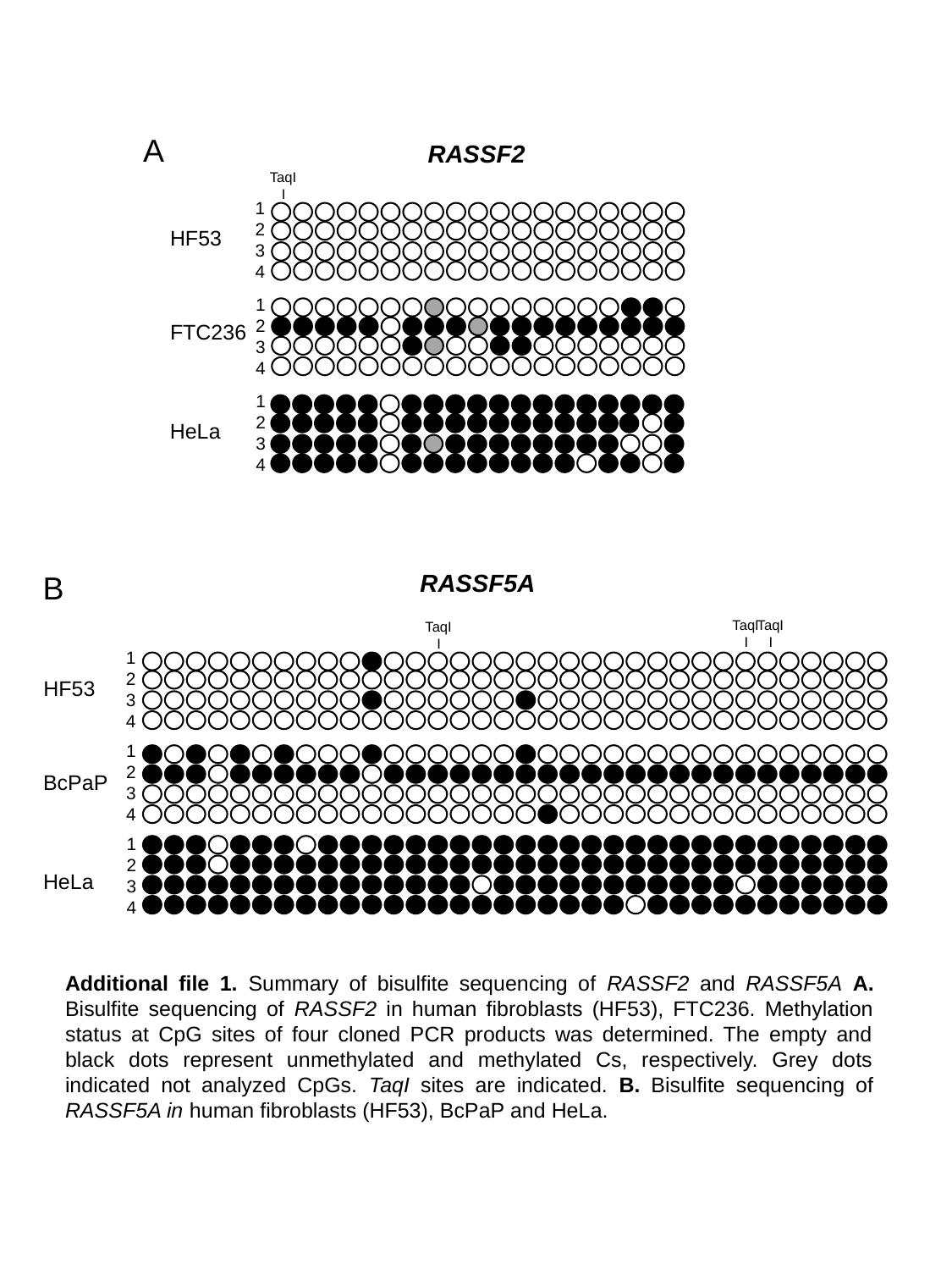

A
RASSF2
TaqI
 I
1
2
3
4
HF53
1
2
3
4
FTC236
1
2
3
4
HeLa
B
RASSF5A
TaqI
 I
TaqI
 I
TaqI
 I
1
2
3
4
HF53
1
2
3
4
BcPaP
1
2
3
4
HeLa
Additional file 1. Summary of bisulfite sequencing of RASSF2 and RASSF5A A. Bisulfite sequencing of RASSF2 in human fibroblasts (HF53), FTC236. Methylation status at CpG sites of four cloned PCR products was determined. The empty and black dots represent unmethylated and methylated Cs, respectively. Grey dots indicated not analyzed CpGs. TaqI sites are indicated. B. Bisulfite sequencing of RASSF5A in human fibroblasts (HF53), BcPaP and HeLa.
