# Supplementary material for: Frequent epigenetic inactivation of RASSF2 in thyroid cancer and functional consequences
Source: Mol Cancer. 2010 Sep 29;9:264. doi: 10.1186/1476-4598-9-264 (PMC2956732; doi:10.1186/1476-4598-9-264)
Supplement: Additional file 2 — Quantitative interaction analysis in the ONPG assay. Graph of the quantitative yeast two-hybrid interaction results [file 1476-4598-9-264-S2.PPT]

## Slide 1
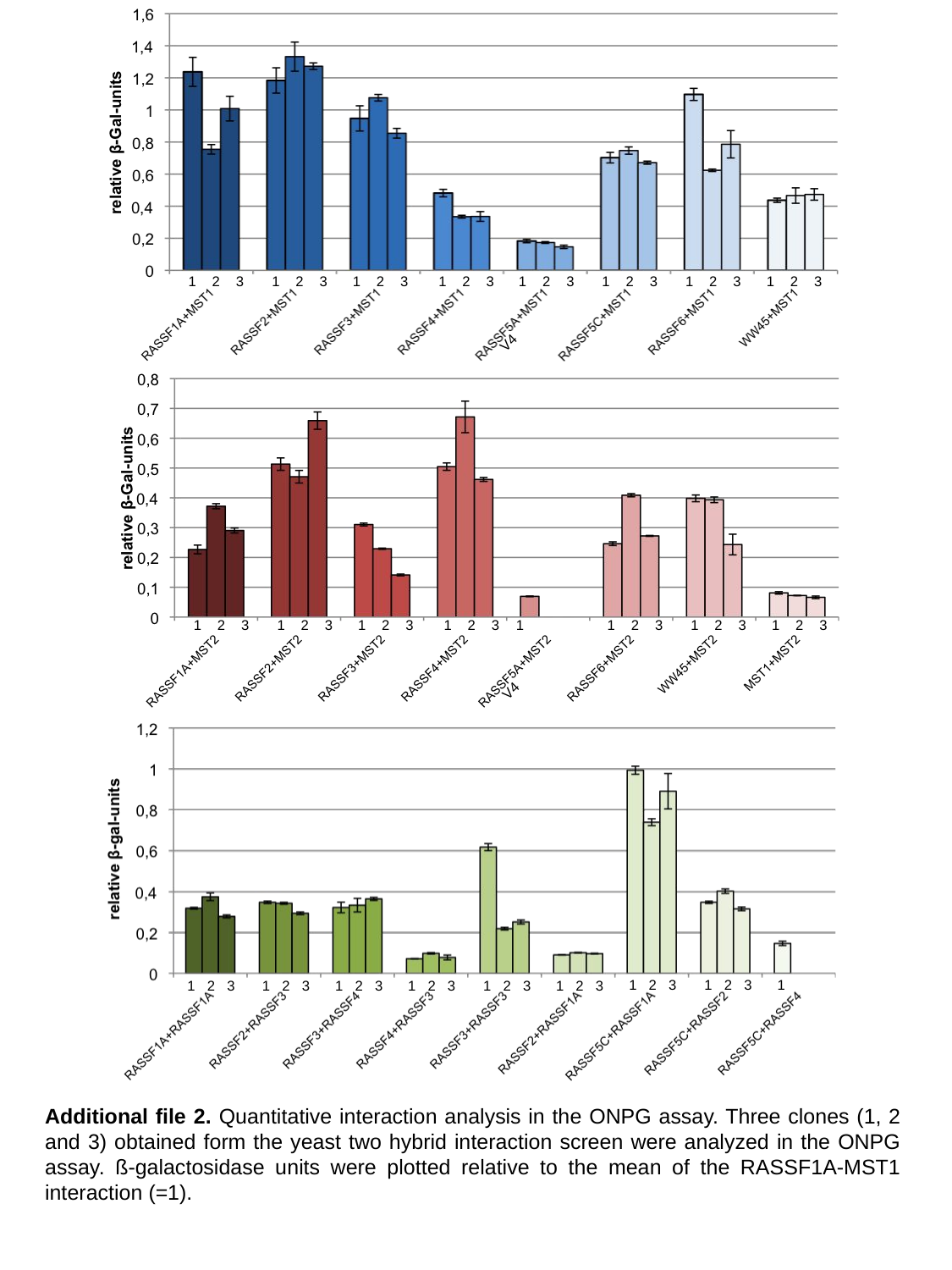

1 2 3
1 2 3
1 2 3
1 2 3
1 2 3
1 2 3
1 2 3
1 2 3
V4
1 2 3
1 2 3
1 2 3
1 2 3
1
1 2 3
1 2 3
1 2 3
V4
1 2 3
1 2 3
1
1 2 3
1 2 3
1 2 3
1 2 3
1 2 3
1 2 3
Additional file 2. Quantitative interaction analysis in the ONPG assay. Three clones (1, 2 and 3) obtained form the yeast two hybrid interaction screen were analyzed in the ONPG assay. ß-galactosidase units were plotted relative to the mean of the RASSF1A-MST1 interaction (=1).
