# Supplementary material for: Frequent epigenetic inactivation of RASSF2 in thyroid cancer and functional consequences
Source: Mol Cancer. 2010 Sep 29;9:264. doi: 10.1186/1476-4598-9-264 (PMC2956732; doi:10.1186/1476-4598-9-264)
Supplement: Additional file 3 — Binding analysis of RASSF2ΔSARAH and MST1 in co-precipitation. Figure of Western blot [file 1476-4598-9-264-S3.PPT]

## Slide 1
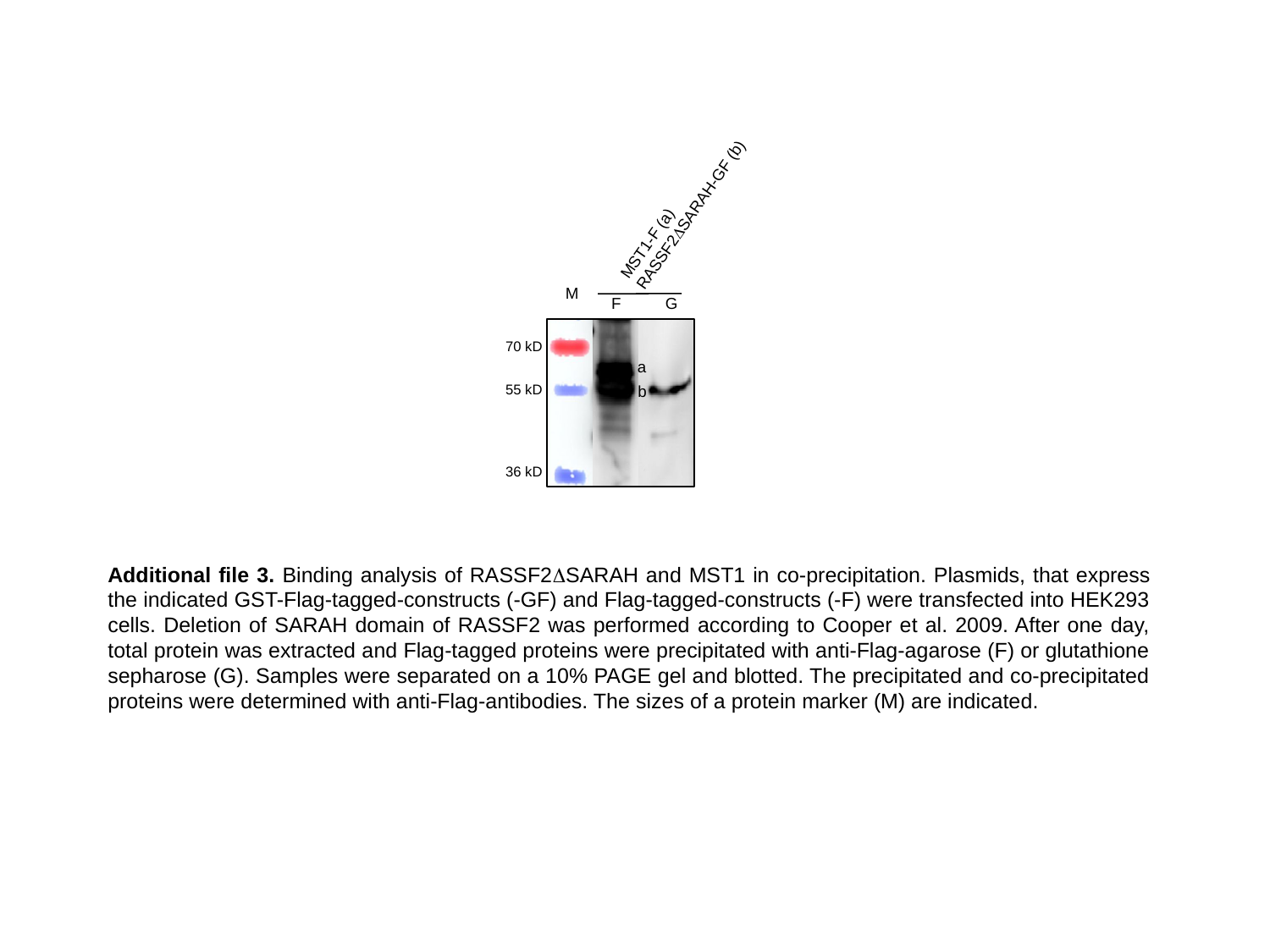

MST1-F (a)
RASSF2ΔSARAH-GF (b)
M
F G
70 kD
a
55 kD
b
36 kD
Additional file 3. Binding analysis of RASSF2ΔSARAH and MST1 in co-precipitation. Plasmids, that express the indicated GST-Flag-tagged-constructs (-GF) and Flag-tagged-constructs (-F) were transfected into HEK293 cells. Deletion of SARAH domain of RASSF2 was performed according to Cooper et al. 2009. After one day, total protein was extracted and Flag-tagged proteins were precipitated with anti-Flag-agarose (F) or glutathione sepharose (G). Samples were separated on a 10% PAGE gel and blotted. The precipitated and co-precipitated proteins were determined with anti-Flag-antibodies. The sizes of a protein marker (M) are indicated.
