# Supplementary material for: Frequent epigenetic inactivation of RASSF2 in thyroid cancer and functional consequences
Source: Mol Cancer. 2010 Sep 29;9:264. doi: 10.1186/1476-4598-9-264 (PMC2956732; doi:10.1186/1476-4598-9-264)
Supplement: Additional file 4 — RASSF2 induced apoptosis. Figure of TUNEL assay. [file 1476-4598-9-264-S4.PPT]

## Slide 1
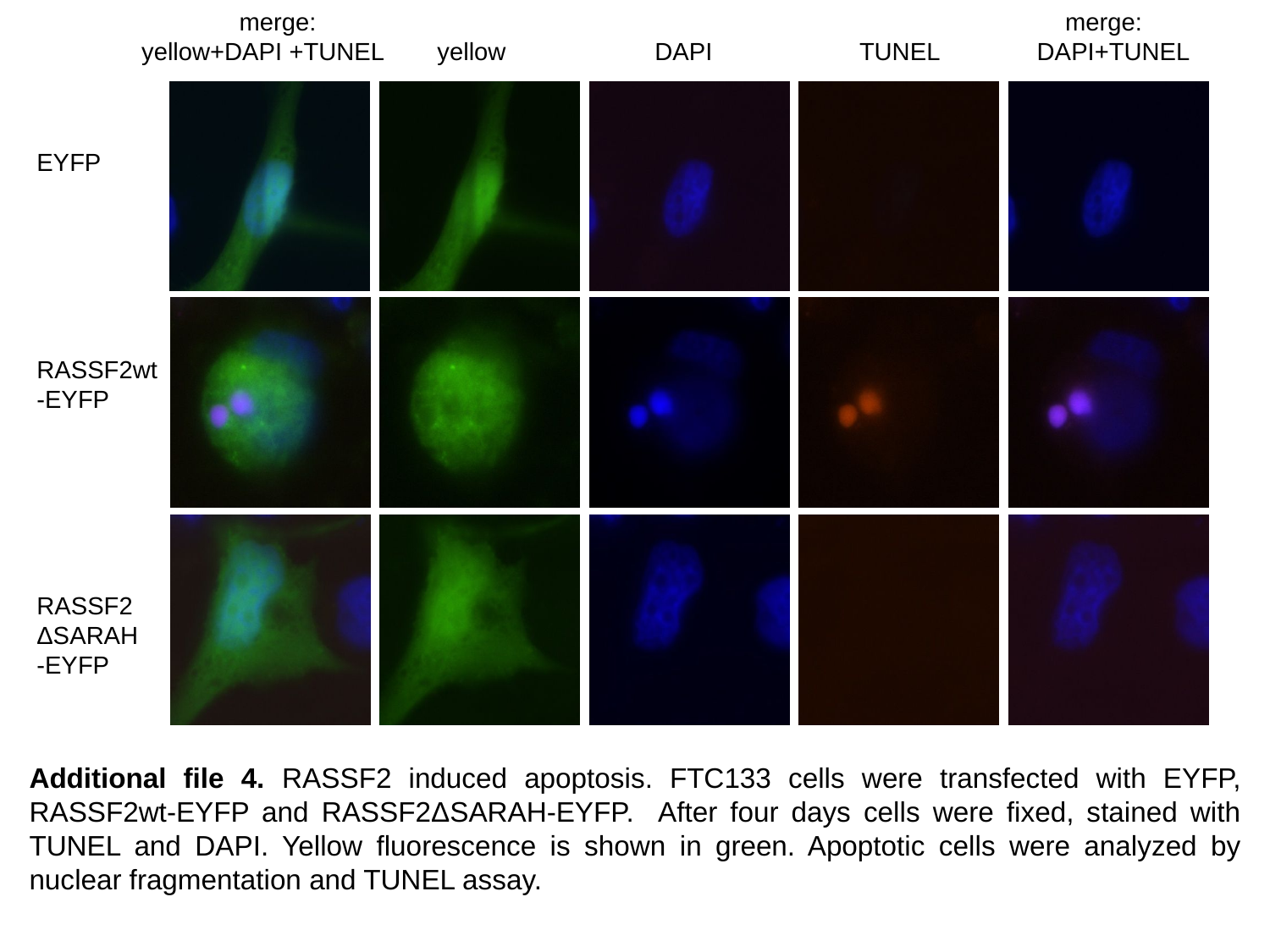

merge: 	 					 merge:
yellow+DAPI +TUNEL	 yellow	 DAPI	 TUNEL DAPI+TUNEL
EYFP
RASSF2wt
-EYFP
RASSF2
ΔSARAH
-EYFP
Additional file 4. RASSF2 induced apoptosis. FTC133 cells were transfected with EYFP, RASSF2wt-EYFP and RASSF2ΔSARAH-EYFP. After four days cells were fixed, stained with TUNEL and DAPI. Yellow fluorescence is shown in green. Apoptotic cells were analyzed by nuclear fragmentation and TUNEL assay.
