# Supplementary material for: Frequent epigenetic inactivation of RASSF2 in thyroid cancer and functional consequences
Source: Mol Cancer. 2010 Sep 29;9:264. doi: 10.1186/1476-4598-9-264 (PMC2956732; doi:10.1186/1476-4598-9-264)
Supplement: Additional file 5 — Primer sequences and conditions for COBRA. Table of oligonucleotides used for methylation analysis [file 1476-4598-9-264-S5.DOC]

| **gene** | **PCR** | **upper primer** | **lower primer** | **product (bp)** | **T_a_ in °C** | **cycles** |
| --- | --- | --- | --- | --- | --- | --- |
| RASSF2 | 1.PCR | 5‘GGTGTAGGGTTGGGGAGGGTTTGAT 3‘ | 5‘AACAAAACCCTCAATCTCCCTATAAAACCA 3‘ | 384 | 60 | 25 |
|  | 2.PCR |  | 5‘CCCAACCACCTCAAACACCAACTCC 3‘ | 220 | 60 | 35 |
| RASSF3 | 1.PCR | 5‘GGGTAATTTTTATTTATTTTTATAGGATTTTG 3‘ | 5‘ACCCCGCCTCCCTAAACCCC 3‘ | 366 | 60 | 45 |
| RASSF4 | 1.PCR | 5‘AAATGTTTTGGGTTTTATTGATAAGTTTTTTT 3‘ | 5‘ACCAAATATCCCCAAACCATTATTCAACTC 3‘ | 275 | 58 | 35 |
|  | 2.PCR | 5‘GTAGCGGTTTTTGTTGGAAGTTTAGGAGTT 3‘ |  | 175 | 56 | 30 |
| RASSF5A | 1.PCR | 5‘GGATAGTTTTGTTAGTTTTTGGAGGTATT 3‘ | 5‘ACCCTAAACCTTCAACCCTACCTCTT 3‘ | 363 | 56 | 25 |
|  | 2.PCR |  | 5‘CTTACCAATCACTTTCCCCAACAC 3‘ | 334 | 56 | 35 |
| RASSF5C | 1.PCR | 5‘GGTTTTGAGGAATTTTGTAGAGGAA 3‘ | 5‘AAAAAAAATAAACACCCCTCCCC 3‘ | 380 | 53 | 35 |
|  | 2.PCR | 5‘AGGAAGTGGTTTTAGAATTGTTTTA 3‘ | 5‘TAAACCCCTAACTCTAAACCCC 3‘ | 323 | 50 | 25 |
| WW45 | 1.PCR | 5‘TGGGGGGAAAGAGAGTTATTGTTTT 3‘ | 5‘AACCAAAACCAAAACCATAATCC 3‘ | 421 | 57 | 20 |
|  | 2.PCR | 5‘GTTGAGGATGAGTGAGGATAGT 3‘ |  | 192 | 55 | 30 |
| MST1 | 1.PCR | 5‘TTTGGTTTGTTAAGAATTGTTAGTTTTTTT 3‘ | 5‘TCCTCTTAACCAATAACCCCTCAC 3‘ | 464 | 55 | 25 |
|  | 2.PCR | 5‘GTTTGTGAAATGGGATTTAGGATTTAGG 3‘ |  | 410 | 56 | 30 |
| MST2 | 1.PCR | 5‘GGGGGTGGTTAGGTTAGGTTTTGTTG 3‘ | 5‘AACACCTATCAAAACAACACATCCACAA 3‘ | 294 | 57 | 20 |
|  | 2.PCR |  | 5‘ACTATATAACTATTAATATTTAATTCCTCCC 3‘ | 256 | 55 | 30 |

**Additional file 5**. Primer sequences and conditions for COBRA
