# Supplementary material for: Frequent epigenetic inactivation of RASSF2 in thyroid cancer and functional consequences
Source: Mol Cancer. 2010 Sep 29;9:264. doi: 10.1186/1476-4598-9-264 (PMC2956732; doi:10.1186/1476-4598-9-264)
Supplement: Additional file 6 — Primer sequences and conditions for qRT-PCR. Table of oligonucleotides used for expression analysis [file 1476-4598-9-264-S6.DOC]

**Additional file 6**. Primer sequences and conditions for qRT-PCR

| **gene** | **upper Primer** | **lower Primer** | **product (bp)** | **T_a_ [°C]** | **cycles** |
| --- | --- | --- | --- | --- | --- |
| RASSF2 | 5‘TCCTCCAGGGCCCATGTGAGC 3‘ | 5‘TTGCTGGGGTCTCGGCTATCTCC 3‘ | 237 | 60 | 34 |
| RASSF5A | 5’GCACCCTCACCGTGACCTTCAGC 3‘ | 5’CCGCCGGAGTTTCAGATGCATT 3‘ | 191 | 60 | 28 |
| RASSF5C | 5‘TGCAGCCTGGACGAGGAACTGG 3‘ | 5’CCGCCGGAGTTTCAGATGCATT 3‘ | 252 | 60 | 30 |
| β-Actin | 5‘CCTTCCTTCCTGGGCATGGAGTC 3‘ | 5’CGGAGTACTTGCGCTCAGGAGGA 3‘ | 226 | 60 | 25 |
